# Developing Efficient Methods of Sperm Cryopreservation for Three Fish Species (*Cyprinus carpio* L., *Schizothorax prenanti*, *Glyptosternum maculatum*)

**DOI:** 10.3390/ijms26104648

**Published:** 2025-05-13

**Authors:** Zheng Zhu, Jingting Yao, Linghui Zeng, Ke Feng, Chaowei Zhou, Haiping Liu, Wanliang Wang, Jianshe Zhou, Hongyan Xu

**Affiliations:** 1 Key Laboratory of Freshwater Fish Reproduction and Development, Ministry of Education, Key Laboratory of Aquatic Sciences of Chongqing, Integrative Science Center of Germplasm Creation in Western China (CHONGQING) Science City, College of Fisheries, Southwest University, Chongqing 402460, China; zz13906334209@163.com (Z.Z.); 13262973352@163.com (J.Y.); zenglinhui99@163.com (L.Z.); fengke127@163.com (K.F.); zcwlzq666@163.com (C.Z.); luihappying@163.com (H.L.); 2 Institute of Aquatic Sciences, Xizang Autonomous Region Academy of Agricultural and Animal Husbandry Sciences, Lasa 850032, China; qlxlsylzfyzx@163.com (W.W.); zjianshe@163.com (J.Z.)

**Keywords:** cryopreservation, sperm viability, DNA fragmentation index, mitochondrial membrane potential, ultrastructure of sperm, fish

## Abstract

Sperm cryopreservation is helpful for maintaining the genetic diversity of fish species. This study was aimed at developing efficient methods to cryopreserve the sperm of three fish species, including koi carp (*Cyprinus carpio* L.), Ya fish (*Schizothorax prenanti*), and *Glyptosternum maculatum*. Firstly, based on the analysis of sperm viability, the cryomedium, dilution ratio, volume, and cooling procedure were assessed and optimized in koi carp. The results showed that the highest sperm viability was up to 63.23 ± 1.36% after a 14-day cryopreservation using the optimal method, briefly, sperm frozen with a volume of 50 μL (Vol.sperm:Vol.cryomedium = 1:9) of cryomedium containing 10% DMSO and 3% sucrose in D17 through ultrarapid cooling. Secondly, both the mitochondrial membrane potential and the DNA fragmentation index of sperm were examined and found to be significantly damaged after the cryopreservation. Intriguingly, the fertilization rate of sperm after 14-day cryopreservation is up to 63.03 ± 1.36% and the elongation of cryopreservation time (210 days) just slightly affected the fertilization rate (55.09 ± 4.70%) in koi carp. Thirdly, the optimal cryopreservation method was applied to cryopreserve *Glyptosternum maculatum* sperm; the cell viability was 45.39 ± 4.70%. And then this method, after a minor modification (3% sucrose of cryomedium replaced with 3% SMP) was adopted to cryopreserve Ya fish sperm, the cell viability was up to 70.45 ± 2.23%. Lastly, the ultrastructure and morphology of sperm was observed by SEM, and it was found that the cryopreservation prominently caused sperm head swelling and tail shortening in three fish species. In conclusion, this study established effective methods for cryopreserving sperm in three fish species and elaborated the injuries on sperm caused by cryopreservation. And the findings facilitate developing more protocols with practical value to cryopreserve sperm in different fish species.

## 1. Introduction

Sperm cryopreservation is a key technique for reproductive management, stock enhancement, cellular engineering breeding, and genetic preservation, using different cryomedium and processes to improve sperm viability after long-term freezing. The viability of sperm has been proven to be associated with a variety of factors, such as the physiological biochemical characters of sperm, the components of cryomedium, and the cooling procedure [1]. It is well-known that the quality of frozen–thawed sperm varies significantly among different species. In human, the sperm frozen in liquid nitrogen (LN) for 40 years has been proven to be safe and effective for fertility [2]. In cattle, the ratio of mobile frozen–thawed sperm was greater than 50%, slightly higher than in pig, while, in sheep, it was around 30%~40%, and, in horses, it was only ~30% [3]. In aquatic species, a great number of encouraging results have been reported. In a study of beluga sturgeon (*Huso huso*), both a high sperm motility (69%) and fertilization rate (72%) were documented when the sperm was frozen at a cooling rate of −40 °C/min for 30 days [4].

The addition of ascorbic acid to cryomedium could improve the ratio of mobile frozen–thawed sperm to 50% in rainbow trout (*Oncorhynchus mykiss*) [5]. Nowadays, dimethyl sulfoxide (DMSO), methanol (MeOH), and glycerin have been used as the permeable cryoprotectants for sperm cryopreservation in mammals and fishes, and sucrose, glucose, trehalose, fetal bovine serum (FBS), bull serum albumin (BSA), and milk powder are frequently considered as effective non-permeable cryoprotectants [6,7,8,9]. In previous studies, the effects of two main cooling procedures on the frozen–thawed sperm (in fish and mammals) quality were extensively assessed and compared, including slow cooling and ultrarapid cooling (where samples were hung above the liquid nitrogen and cooled by the vapor of liquid nitrogen (LN)); however, the results were not always consistent [7,8]. Compared with the slow-cooling procedure, the ultrarapid-cooling procedure was easier and provided a higher efficiency of vitrification; thus, it could improve the membrane integrity and the ratio of mobile frozen–thawed sperm [10].

Although sperm cryopreservation technology is becoming increasingly easy, at present, in many species, this technology can still cause lots of damage to sperm. For example, a study on sheep (*Ovis aries*) revealed that sperm cryopreservation caused damage to the DNA integrity and mitochondrial membrane activity of sperm [11]. Cryoinjuries are almost inevitable in cellular cryopreservation, mainly induced by ice crystal formation and cold shock. During freezing, the induction of ice crystal formation and cold shock result in the destruction of organelles, such as cytoskeleton proteins, DNA, mitochondria, acrosome, and plasma membrane, then damaging the cellular structures [1]. Cooling also caused oxidative stress and an excessive amount of reactive oxygen species (ROS), which in turn induced membrane lipid peroxidation and damage to mitochondria and DNA [12]. Furthermore, inappropriate cryomedium and/or equilibrium procedures, such as the freezing and thawing processes, can cause cytotoxicity and osmotic shock [13]. Conventionally, the motility, viability, fertilization rate, and hatching rate are evaluated, and these indexes are usually closely associated with the efficiency of sperm cryopreservation. For the assessment of sperm motility, sperm should be activated by activator solution, observed by microscope, and analyzed with CASA (computer-assisted sperm analysis) [14]. Trypan blue staining and acridine orange/propidium iodine (AO/PI) staining are always used for cells’ viability assessment, and the fertilization rate and hatching rate are also the important indexes evaluated [6]. The injury mechanism behind cells’ freezing has been documented by a great amount of research, and the evaluated indexes always included the organelles’ damage (e.g., cell membrane, acrosome, and mitochondria), DNA integrity, superoxide (e.g., lipid peroxidation, reactive oxygen species, malondialdehyde), and antioxidant enzymes (e.g., superoxide dismutase, peroxidase) [6].

The three fish species studied, koi carp, Ya fish, and *Glyptosternum maculatum*, all have high economic value [15,16,17]. Koi carp, originating in Japan, is now popular worldwide and highly prized for its ornamental value. Ya fish, a partial migratory fish endemic to China, is mainly distributed in the upper reaches of the Yangtze River, including the Jinsha River, Dadu River, Minjiang River, and Qingyi River. *Glyptosternum maculatum* is mainly distributed in the Brahmaputra River basin in Tibet, China, and the Brahmaputra River in India. This species, an important indigenous economic fish in Tibet, has high nutritional value but is difficult to breed artificially due to its long maturation cycle of gonads and low reproductivity.

Here, a comprehensive experimental approach was designed to optimize the protocol of sperm cryopreservation for koi carp, Ya fish, and *Glyptosternum maculatum*. For each species, different extenders (such as D17, Kurokura1, HBSS300 for koi carp; D17 and CCES2 for Ya fish), permeable cryoprotectants (DMSO, MeOH for koi carp; DMSO, MeOH, glycerol for Ya fish), non-permeable cryoprotectants (sucrose, D-trehalose, FBS, BSA for koi carp; sucrose, D-trehalose, SMP, BSA for Ya fish), dilution ratios (Vol.sperm:Vol.cryomedium = 1:4, 1:9, 1:19 for all three species), and volumes (50 μL, 100 μL, 200 μL, 400 μL for koi carp; 50 μL, 100 μL, 150 μL for Ya fish) were assessed. Additionally, different cooling procedures, including slow and ultrarapid cooling, were evaluated, and the injuries to sperm caused by cryopreservation were also determined, such as the damage to cell membrane integrity, DNA integrity, mitochondrial membrane potential, the ultrastructure, and so on. The aim of this study was to develop practical and efficient cryopreservation methods for the genetic resources of three fish species, which may also serve as references for germplasm cryopreservation in other related fish species.

In recent years, distinguished progress has been achieved in studies on fish cryopreservation. As for the impact of cryopreserved sperm on offspring development, a study was conducted in *Rhamdia quelen*, and the result showed that, although cryopreservation reduced sperm motility and reduced the fertilization rate, there was no significant difference in hatching rates and larval development between the cryopreserved and fresh sperm. This indicated that the cryopreserved sperm of *Rhamdia quelen* could still produce normal larvae, providing key insights for fish genetic conservation and the application of cryopreserved sperm in aquaculture [18]. Moreover, the optimization of sperm cryopreservation techniques has been widely reported in fish species. For instance, a study documented that Hank’s Balanced Salt Solution (HBSS) was the optimal extender for sperm cryopreservation in Qihe Crucian Carp (*Carassius auratus*) [19]. A 15% methanol (MeOH) solution was the best choice for short-term low-temperature storage at 4 °C, and 20% dimethyl sulfoxide (DMSO) was the most suitable for liquid nitrogen cryopreservation [19]. Also, it was found that an ultra-freezer (−80 °C) could not replace liquid nitrogen for the sperm cryopreservation in this species. Additionally, a study reported the effect of melatonin on cryopreserved sperm in brown-marbled grouper. The result showed that adding an appropriate amount of melatonin (MT) to the extender could enhance the motility, mitochondrial membrane potential, and fertilization ability of cryopreserved sperm [20]. Specifically, 0.1–0.25 mg/mL of MT provided the best results, while a concentration of 0.5 mg/mL may be toxic to the cells. In giant grouper (*Epinephelus lanceolatus*), the altered molecules were investigated, and 1911 deferentially expressed genes (DEGs) were identified in the long-term cryopreserved sperm; these genes were significantly enriched in processes such as pilus assembly, metabolic processes, and the MAPK signaling pathway [21]. The study also reported that four key genes, scarb1, odf3, exoc8, and atp5f1d, related to mitochondria and flagella were significantly regulated in cryopreserved sperm. These findings benefit our understanding of the molecular mechanisms behind sperm cryoinjuries and provide an index for developing techniques for long-term sperm cryopreservation [21]. In *Salmo salar*, the impact of dietary fatty acids on fish sperm quality and cryopreservation tolerance was extensively investigated [22]. The study pointed out that the modification of aquafeed formulations, such as replacing fish oil with vegetable oil, could alter the fatty acid profiles of fish tissues and gametes, thereby affecting sperm quality, the success rate of cryopreservation, and the viability of progeny. Fatty acids in the sperm membrane, such as EPA, DHA, and ARA, are crucial for sperm motility and fertilization rates after thawing. Therefore, optimizing the components of cryoprotectant, the freezing procedure, and the diet of broodstock are essential for improving sperm cryotolerance. In a future study, the application of omics technologies is needed to further enhance reproductive outcomes and the sustainability of aquaculture [22].

Generally, many progresses in fish cryopreservation have been achieved, but there is a lack of systemic studies that simultaneously determine the effects of multiple factors, such as sperm cryopreservation, dietary factors, freezing procedures, and others, on offspring development across different fish species. Likewise, the molecular mechanisms underlying sperm cryoinjury are not fully understood. Therefore, in this study, we aim to bridge these gaps with a novel and comprehensive approach. Unlike previous studies that often focus on one or two aspects in isolation, we systematically investigate multiple factors affecting sperm cryopreservation in three distinct fish species: koi carp, Ya fish, and *Glyptosternum maculatum*. We simultaneously evaluate the impact of various cryopreservation parameters (including cryomedium components, dilution ratios, volumes, and cooling procedures) on sperm viability, DNA integrity, mitochondrial membrane potential, and ultra-structure. This multi-faceted approach allows us to develop more effective and species-specific cryopreservation methods.

Furthermore, we explore the transferability of the optimized protocol among different species. By modifying the established protocol for koi carp and applying it to Ya fish and *Glyptosternum maculatum*, we can determine the adaptability of the protocol and gain insights into the similarities and differences in sperm cryopreservation requirements among different fish species. This not only helps in the conservation of the genetic resources of these specific fish but also provides a valuable reference for future research on sperm cryopreservation in other fish species. Overall, our study’s novelty lies in its comprehensive and comparative approach, which is expected to significantly advance the field of fish sperm cryopreservation.

## 2. Results

In this study, the AO/PI (acridine orange/propidium iodide) staining method was used to assess sperm viability (Figure S1), DNA fragmentation was detected using Wright’s staining method, and Rh-123 (Rhodamine 123) staining was conducted to analyze sperm mitochondrial function. Scanning electron microscopy (SEM) was utilized to examine sperm morphology, surface characteristics, and ultrastructure.

### 2.1. Effects of Different Sperm Cryopreservation Protocols on the Sperm Viability in Koi Carp

**Figure 1:**
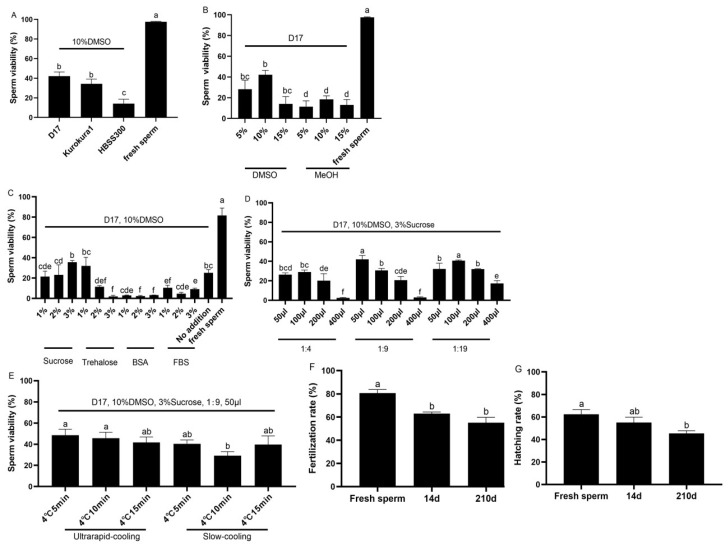
Establishment of sperm cryopreservation protocol in koi carp. After a 14 d freeze in liquid nitrogen, the viability of sperm was assessed by AO/PI staining under microscopy. (**A**) Effects of different extenders on sperm viability. The cryomedium prepared with 10% DMSO being dissolved in four extenders; (**B**) Effects of different permeating cryoprotectants on sperm viability. The cryomedium prepared using D17 added with MeOH or DMSO at different concentrations; (**C**) Effects of different non-permeating cryoprotectants. Serum: BSA, bovine serum albumin; (**D**) Effects of different volumes and dilution ratios of 10% DMSO and 3% sucrose dissolved in D17 for the cryomedium, with fish semen diluted with cryomedium in different volumes and at different dilution ratios; (**E**) Effects of different cooling procedures. Before being frozen, the cryovials were equilibrated at 4 °C for 5–15 min and cooled down via ultrarapid- or slow-cooling procedure; (**F**) Effects of cryopreservation time on the fertilization rate of sperm (14 d, 210 d cryopreserved, and fresh sperm); (**G**) Effects of cryopreservation time on the hatching rate of sperm. All values are presented as the mean ± SEM calculated with the data of nine replicates, different letters indicate the significance of differences (*p* < 0.05). MeOH: methanol; DMSO, dimethyl sulfoxide; FBS, fetal bovine serum; BSA, bovine serum albumin.

In group 1, the sperm was diluted with the extender solutions, D17, HBSS300, and Kurokura1; these solutions can provide an appropriate physiological osmotic pressure and pH [23,24]. In this study, koi carp sperm frozen with D17 had the highest viability (42.18 ± 4.29%), followed by Kurokura1 (34.32 ± 4.85%), and significantly higher than those with HBSS300 (14.19 ± 4.46%) (*p* < 0.05, Figure 1A, Table S1). In Group 2, the sperm was treated with different concentrations (5%, 10%, and 15%) of DMSO and MeOH, since MeOH and DMSO have been widely used as permeable cryoprotectants in the sperm cryopreservation of humans, mice, livestock, and aquatic species [9]. In this study, the viability of frozen–thawed koi carp sperm from the group with cryomedium containing 10% DMSO was up to 42.18 ± 4.29%, significantly higher than those of the 5% DMSO (28.31 ± 8.73%), 15% DMSO (14.19 ± 6.96%), and MeOH groups (*p* < 0.05, Figure 1B, Table S1). In Group 3, impacts of non-permeable cryoprotectants (sucrose, trehalose, BSA, FBS) on sperm viability were various, with sucrose and trehalose posing some positive effects. In this study, the highest viability of sperm was achieved in koi carp using the cryomedium supplemented with 3% sucrose (35.62 ± 1.70%), followed by 1% trehalose (31.94 ± 8.55%) and the control (no supplement of non-permeable cryoprotectant, 25.11 ± 3.35%), while the viability of sperm in protein-treated groups was significantly lower than that of the control group (*p* < 0.05, Figure 1C, Table S1). In Group 4, different dilution ratios (1:4, 1:9, 1:19) and volumes (50 μL, 100 μL, 200 μL, 400 μL) affected sperm viability; koi carp sperm frozen with 1:9 dilution ratio in a volume of 50 μL showed the highest viability (41.98 ± 4.02%), followed by that of sample frozen with 1:19 dilution ratio in volume of 100 μL (40.72 ± 0.66%), and much higher than those of other treatment groups (*p* < 0.05, Figure 1D, Table S1). In Group 5, sperm was frozen with D17, 10% DMSO, and 3% sucrose, with different equilibration times (5 min, 10 min, 15 min) at 4 °C, using either ultrarapid or slow cooling. Ultrarapid and slow cooling are commonly performed in the sperm cryopreservation of mice, livestock, and aquatic species [7,10]. In koi carp sperm frozen though ultrarapid cooling, 5 min of equilibrium time at 4 °C had an excellent viability of 48.62 ± 5.62%, higher than those through 10 min and 15 min equilibrium time of ultrarapid cooling (45.75 ± 5.61% and 41.70 ± 5.20%, respectively) and slow cooling (Figure 1E, Table S1).

### 2.2. Effects of Equilibrium Time on Fertilization Rate and Hatching Rate of Frozen–Thawed Sperm in Koi Carp

The fertilization rate of fresh sperm in koi carp was about 80.71 ± 3.09% and significantly higher than those of frozen–thawed sperm, and that of sperm after 14 d cryopreservation was 63.03 ± 1.36% (*p* < 0.05), while the value after 210 d was 55.09 ± 4.7% (Figure 1F). The hatching rate of fresh sperm was 62.34 ± 4.29%, higher than (*p* < 0.05) sperm frozen for 14 d (55.15 ± 4.69%), and significantly higher than sperm frozen for 210 d (45.34 ± 2.41%) (Figure 1G).

### 2.3. Effects of Different Cryomedia and Procedures on DNA Integrity and Mitochondrial Function of the Frozen–Thawed Sperm in Koi Carp

**Figure 2:**
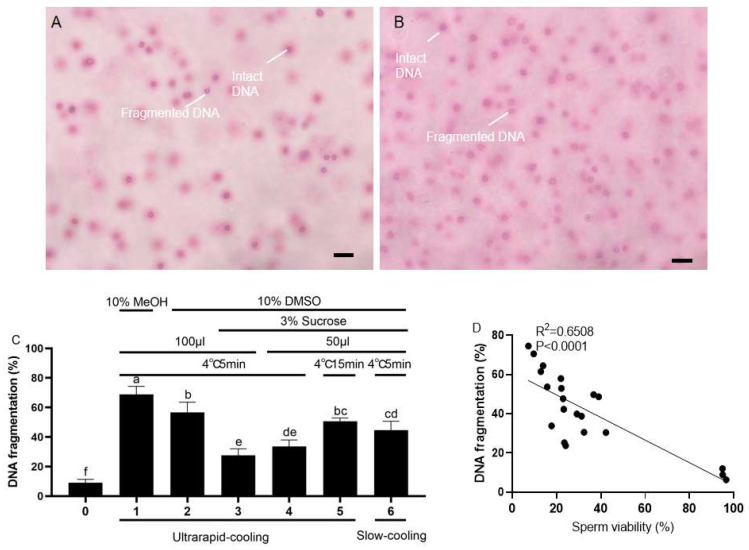
Effects of different cryomedia and procedures on DNA integrity of sperm. (**A**,**B**) The DNA fragmentations of sperm were determined via the wright staining. (**A**) Fresh sperm. (**B**) The frozen–thawed sperm after a 7-day freezing through ultrarapid cooling with cryomedium (50 μL, 1:9) containing 10% DMSO and 3% sucrose in D17. Scale bars, 50 µm. (**C**) The viability of koi carp sperm under different cryopreservation conditions. (**D**) The correlation analysis between the viability of koi carp sperm and the DNA fragmentation index. All values are presented as the mean ± SEM calculated with the data of nine replicates (n = 9). Different letters indicate the significance of differences (*p* < 0.05).

In order to figure out the mechanism behind sperm cryopreservation, DNA integrity (Figure 2A,B) were examined via the wright staining among different groups, including (0) fresh sperm, (1–6) cryopreserved sperm of Groups 1–5. The DNA integrity of sperm in all groups were significantly injured (Figure 2C). Sperm frozen with 100 μL cryomedium containing 10% DMSO and 3% sucrose, after being pre-cooled at 4 °C for 5 min and then ultrarapid-cooled with LN vapor, showed the lowest DNA fragmentation index (27.62 ± 4.44%) among all treatments. On the contrary, the group with 10% MeOH had the highest DNA fragmentation index (68.86 ± 5.45%), followed by the 10% DMSO group (56.70 ± 6.90%) and the group being pre-cooled at 4 °C for 15 min and ultrarapid-cooled (50.74 ± 2.15%, using cryomedium containing 10% DMSO and 3% sucrose) (*p* < 0.05, Figure 2C). It was also found that there was a negative correlation between the DNA fragmentation index and the viability of koi carp sperm after cryopreservation (Figure 2D).

**Figure 3:**
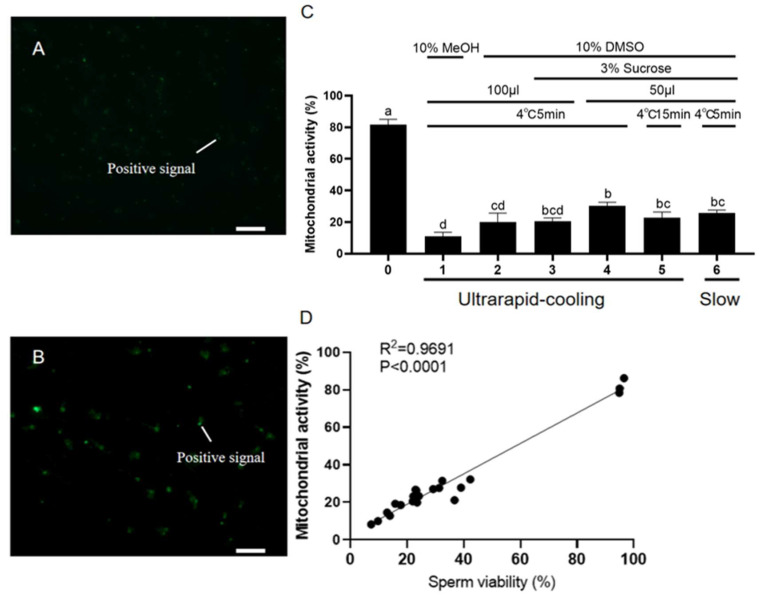
Effects of different cryomedia and procedures on mitochondrial function of sperm. (**A**,**B**) The mitochondrial function of sperm was examined by Rh-123 staining (green, the positive signals indicated by straight lines). (**A**) Fresh sperm. (**B**) The frozen–thawed sperm after a 7-day freezing through ultrarapid-cooling with cryomedium (50 μL), containing 10% DMSO and 3% sucrose, diluted by D17 (sperm diluted at 1:9). Scale bars, 50 µm. (**C**) The mitochondrial activity (the number of positive cells/the total number of cells) of koi carp sperm under different cryopreservation conditions. (**D**) The correlation analysis between the viability of koi carp sperm and the mitochondrial activity. All values are presented as the mean ± SEM calculated with the data of nine replicates (n = 9). Different letters indicate the significance of differences (*p* < 0.05).

The mitochondrial function of sperm was examined by Rh-123 staining (Figure 3A,B). As for the mitochondrial function, sperm frozen with 50 μL of cryoprotectant containing 10% DMSO and 3% sucrose, after being pre-cooled at 4 °C for 5 min and then ultrarapid-cooled, exhibited the highest mitochondrial membrane potential (30.25 ± 2.27%) among all treatments. This value was slightly higher than that of the slow-cooling group (25.70 ± 1.83%) and the group pre-cooled at 4 °C for 15 min before undergoing ultrarapid cooling (22.72 ± 3.66%, with the same cryoprotectant), and was significantly higher than that of other 100 μL groups (*p* < 0.05, Figure 3C). Also, it was also shown that there was a positive correlation between the mitochondrial activity and the viability of koi carp sperm after cryopreservation (Figure 3D).

### 2.4. The Optimized Cryopreservation Protocol Validated in Another Batch of Sperm from Three Fish Species

**Figure 4:**
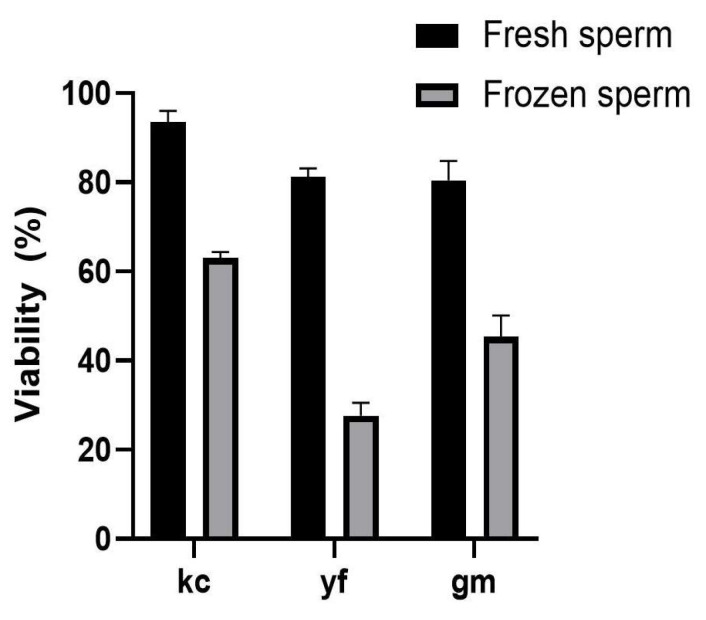
The validation of the sperm cryopreservation protocol established in koi carp. The optimal protocol established was adopted to cryopreserve sperm from another batch of koi carp and two other fish species, Ya fish and *Glyptosternum maculatum* (Regan). kc: koi carp; yf: Ya fish; gm: *Glyptosternum maculatum* (Regan). With the fresh sperm as control, the sperm viability was analyzed by AO/PI staining after a 14 d freeze in liquid nitrogen. All values are presented as the mean ± SEM calculated with the data of nine replicates (n = 9).

Sperm from three fish species (koi carp, Ya fish, and *Glyptosternum maculatum*) were cryopreserved for 14 days using the optimized cryopreservation protocol established in koi carp (10% DMSO, D17, 3% sucrose, 50 μL, 1:9 ratio, equilibration at 4 °C for 5 min, and equilibration 10 cm above liquid nitrogen for 15 min). The effects of cryopreservation on sperm viability were distinct among three species. In koi carp, fresh sperm had a viability of 93.54 ± 2.47%, while cryopreserved sperm showed a viability of 63.03 ± 1.36%. In *Glyptosternum maculatum*, the viability of fresh sperm was 83.09 ± 1.75% and the viability of cryopreserved sperm was 45.39 ± 4.70%. In Ya fish sperm, a great effect caused by cryopreservation, fresh sperm had a viability of 81.34 ± 1.79%, while cryopreserved sperm showed a viability of 27.60 ± 2.96% (Figure 4, Table S2).

### 2.5. Cryopreservation Protocol for Sperm Modified in Ya Fish

**Figure 5:**
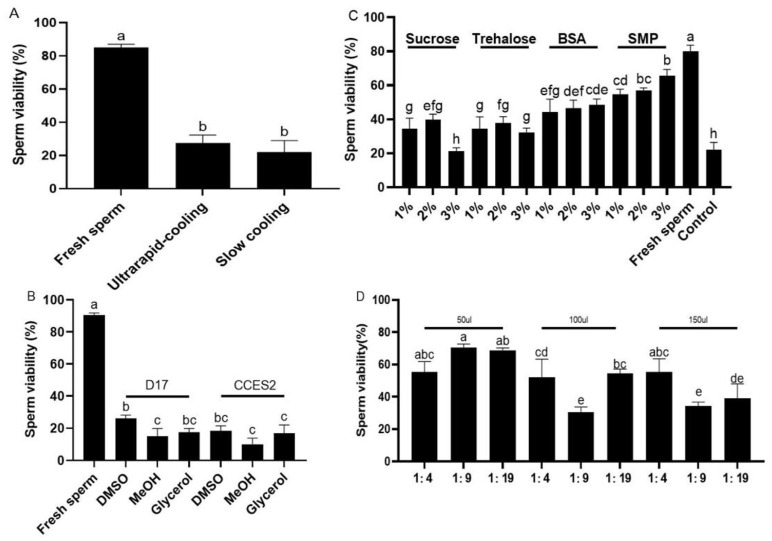
Establishment of sperm cryopreservation protocol in Ya fish. Viability of Ya fish sperm under different cryopreservation conditions. (**A**) Cooling procedures: sperm were cryopreserved through ultrarapid cooling or slow- cooling; (**B**) Different permeating cryoprotectants, sperm were frozen with the cryomedium, D17, or CCES2 containing MeOH, DMSO, or glycerol at 10% concentrations; (**C**) Different non-permeating cryoprotectants; sperm were frozen with 10% DMSO in D17, complemented with sucrose, trehalose, BSA, and SMP at different concentrations of 1%, 2%, and 3%; (**D**) Different volumes and dilution ratios; sperm were cryopreserved with D17 containing 10% DMSO and 3% SMP, in different volumes (50 μL, 100 μL, and 150 μL) and at different dilution ratios (sperm diluted at 1:4, 1:9, and 1:19). All values are presented as the mean ± SEM calculated with the data of nine replicates (n = 9); Different letters indicate the significance of differences (*p* < 0.05). MeOH: methanol; DMSO, dimethyl sulfoxide; SMP, skimmed milk powder; BSA, bovine serum albumin.

Firstly, sperm was treated with D17, 10% DMSO, and 3% sucrose, equilibrated at 4 °C for 5 min, and then ultrarapid- or slow-cooled. In Ya fish, sperm frozen through ultrarapid cooling showed a higher viability than those that underwent slow cooling (22.01 ± 6.91%) (Figure 5A, Table S3). Secondly, the sperm was diluted by D17 or CCES2, with 10% DMSO, MeOH, or glycerol added simultaneously. In this study, the viability of frozen–thawed Ya fish sperm from the group with cryomedium containing 10% DMSO in D17 was up to 26.00 ± 2.10%, significantly higher than those of the 10% MeOH in D17 (15.01 ± 4.66%), 10% MeOH in CCES2 (10.01 ± 3.89%), and 10% MeOH in glycerol (16.89 ± 5.13%) groups (*p* < 0.05, Figure 5B, Table S3). Thirdly, different non-permeable cryoprotectants (sucrose, trehalose, BSA, SMP) have different impacts on sperm viability. In this study, the highest viability of sperm was achieved in Ya fish with cryomedium supplemented with 3% SMP (65.62 ± 3.79%), followed by 2% SMP (57.13 ± 1.40%) and the control (no supplement of non-permeable cryoprotectant, 20.04 ± 4.44%); the viability of sperm in carbohydrates-treated groups was significantly lower than that of the control group (*p* < 0.05, Figure 5C, Table S3). Fourthly, different dilution ratios (1:4, 1:9, 1:19) and volumes (50 μL, 100 μL, 150 μL) of the cryopreservation also affect sperm viability. In Ya fish, sperm frozen with a dilution ratio of 1:9 in a volume of 50 μL showed the highest viability (70.45 ± 2.23%), followed by that of the sample frozen with 1:19 dilution ratio in a volume of 50 μL (68.78 ± 1.45%), and much higher than those of other groups (*p* < 0.05, Figure 5D, Table S3).

### 2.6. Effects of Cryopreservation on Sperm Ultrastructure

**Figure 6:**
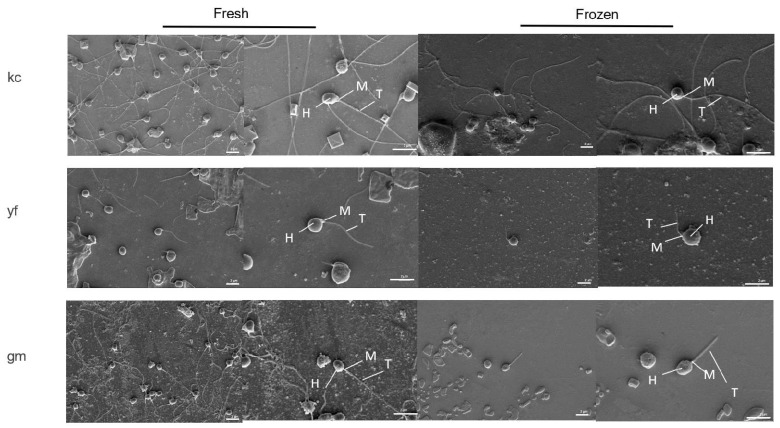
The ultrastructural morphology of fresh and frozen–thawed sperm. The ultrastructure and morphology of sperm were observed and comparatively analyzed among three fish species by ZEISS EVO 10. kc: koi carp; yf: Ya fish; gm: *Glyptosternum maculatum* (Regan). H: head; M: midpiece; T: tail. Scale bars, 4 μm.

Post-cryopreservation, sperm from three species all showed head swelling (Figure 6) (for example, in koi carp, the head length changed from 1.93 µm to 2.25 µm, and the head width change from 1.62 to 1.96) and significant tail shortening (Figure 6) (for example, in Ya fish, the tail length changed from 8.19 µm to 5.60 µm) (Table 1). Midpiece dimensions varied and obviously contracted in koi carp sperm (from 1.19 µm to 1.00 µm) (Table 1). These results indicate the main damage caused by cryopreservation on sperm, including the disruption of cells’ membranes, alterations to motility structure, and some morphological changes.

Through ZEISS EVO 10, the ultrastructural morphology of fresh and frozen–thawed sperm was comparatively analyzed in three fish species. The head length, head width, midpiece length, midpiece width, tail length, and tail width of sperm from three fish species before and after cryopreservation are shown below.

## 3. Discussion

### 3.1. An Effective Method Developed for Sperm Cryopreservation Using a Sucrose–DMSO Cryomedium in Koi Carp

The successful cryopreservation of sperm largely depends on the choice of extender, playing a critical role in maintaining physiological osmotic pressure, pH, and energy supply and preventing sperm agglutination, bacterial contamination, and oxidative stress [25]. In our study, D17 provided the best result among the extenders tested, giving the highest viability of post-thaw sperm and not activating the fresh sperm. Its superiority is likely due to the higher concentrations of KCl and glucose; these are crucial for maintaining osmolality and supporting metabolic processes in sperm. For instance, K^+^ is essential for sperm motility maintenance during the quiescent stage, and its concentration in D17 aligns with the physiological requirements of common carp sperm (73.01–78.87 mmol/L) [26,27]. In the present study, we found that DMSO as a permeable cryoprotectant was more effective at a suitable concentration of 10% than MeOH. Similar results were documented in various freshwater fishes, including Scombridae fishes, Tambaqui (*Colossoma macropomum*), Blackfin Goodea (*Goodea atripinni*), and rainbow trout [28,29,30,31]. However, many studies have suggested that MeOH is a better permeable cryoprotectant than DMSO, such as in sterlet (*Acipenser ruthenus* L.) and Mozambique tilapia (*Oreochromis mossambicus*) [32,33]. These kinds of discrepancies were probably attributed to the different amount or composition of lipids and proteins in the sperm of different fish species since the lipids and proteins were considered as the main factors injured during cell freezing [34,35,36]. Several research works have reported that MeOH could disrupt the mitochondrial transport system and damage mitochondrial DNA, which may partially explain the lower mitochondrial activity and viability that occurred in the sperm of MeOH-treated groups than that in other treatment groups [37,38].

Research has shown that, in the cryopreservation of chicken sperm, low-concentration (1 mM) sucrose exhibits significant advantages. Sucrose not only significantly enhanced the membrane integrity, acrosome integrity, and mitochondrial function of frozen -thawed sperm, but also led to a fertility rate as high as 91%, which was significantly higher than the 86% in the control group [39]. Likewise, 0.25 mol/L sucrose was considered as an optimal concentration for sperm vitrification in humans [40]. The results of our study also proved that sucrose is a useful supplement for koi carp sperm cryopreservation. A study demonstrated that adding 1.5% skimmed milk powder (SMP) into the cryomedium could significantly improve the viability and motility of cryopreserved sperm in Hu sheep [41]. Similarly, the results of our study also proved that SMP was a useful supplement for sperm cryopreservation in Ya fish. SMP, as a non-permeable cryoprotectant, has a protective effect on zebrafish sperm cells during the cryopreservation process [42]. It can increase the proportion of sperm with normal morphology, protect the integrity of the cell membrane, and reduce oxidative damage and DNA damage in cryopreserved cells [42]. SMP could also increase the survival rate of the cryopreserved sperm of Ya fish. This may be attributed to the potency of SMP in protecting cell membrane integrity, improving cell anti-lipid peroxidation, providing cell with nutritional support, stabilizing protein structure, and regulating osmotic pressure in cells.

### 3.2. The Dilution, Density, and Volume of Sperm Are Important for Cryopreservation

On the premise of the same quality, a higher density of sperm in a vessel is more likely to inseminate more eggs. Sperm dilution ratios normally range from 1:1 to 1:20, and are different by species. In studies of Atlantic croaker (*Micropogonias undulatus* L.), the motility parameter of sperm samples diluted at 1:3 was significantly higher than that of undiluted sperm [43]. The same results were also observed in Atlantic sturgeon (*Acipenser oxyrinchus*) and haddock (*Melanogrammus aeglefinus*) [44,45]. Meanwhile, in freshwater species, the dilution ratios of sperm are generally high. The dilution ratios of 1:9 or 1:10 were successfully applied in the short-term storage of African catfish, perch spermatozoa (*Perca fluviatilis* L.), and freshwater streaked prechilled (*Prochilodus lineatus*) [46,47]. In a word, the dilution ratios must be tailored for different fish species. In the present study, 1:9 was the best dilution ratio: the viability of sperm diluted at 1:9 was significantly higher than that of a low dilution ratio (1:4) or a high one (1:19). Ciereszko et al. (2013) supposed that higher dilution ratio could benefit the contact between extender and sperm, thereby accelerating the permeation of cryoprotectants [48]. But Viveiros and Godinho (2009) hypothesized that seminal plasma may have a positive effect on the energy utilization and ionic balance in sperm, and the ultra-dilution of sperm using extenders would reduce the concentration of potential active ingredients in seminal plasma, thereby reducing sperm motility [49]. Therefore, keeping a balance between an appropriate level of cryoprotectants and seminal plasma is of importance.

### 3.3. The Advantages of Ultrarapid Cooling in Cryopreserving Fish Sperm

Vapor (liquid nitrogen) immersion is a technique widely used in sperm cryopreservation. Recently, slow cooling, vitrification, and ultrarapid cooling have been assessed and comparatively analyzed in the sperm cryopreservation of some species, including humans, honey bees, bovine (*Apis mellifera* L.), and fallow deer (*Dama dama*) [50,51,52]. These results were inconsistent because of different species and freezing devices, but the advantage of ultrarapid cooling has been commonly accepted, such as time-saving and a low cost of equipment. In aquatic species, ultrarapid cooling has been widely applied in sperm cryopreservation and provided successful fertilization tests [33,53,54]. In the present study, the advantage of vapor immersion was also proven by the higher viability and lower DNA damage of sperm. The equilibrium at 4 °C of sperm in the interval step between mixing sperm with cryoprotectants and dropping cryotubes into liquid nitrogen is expected to be beneficial, as it allows a better osmotic balance after sperm interact with a cryoprotectant. However, in the present study, the sperm viability was decreased with the elongation of equilibrium time at 4 °C. This may be due to the toxicity of DMSO to sperm; the high DNA damage that occurred in sperm has also provided important evidence for the toxicity of DMSO [55]. As an energy-providing organelle, mitochondrial membrane potential was closely correlated with sperm viability, while DNA integrity was also a critical factor for sperm viability in this study (Figure 2D and Figure 3D).

### 3.4. The Main Injuries on Fish Sperm Caused by Cryopreservation

In the present study, mitochondrial membrane potential is considered as a key index for sperm performance; it was prominently affected by cryopreservation in various fish species such as European seabass (*Dicentrarchus labrax*), sterlet (*Acipenser ruthenus*), common carp, salmon trout (*Oncorhynchus mykiss*), brook trout (*Salvelinus fontinalis*) and sea bream (*Sparus aurata*) [56,57]. After being thawed, cryodamage occurred in the middle piece of the sperm, showing protuberances or plasma membrane thickening due to the loss of the dense sheath surrounding the mitochondria [6,58]. These changes resulted in the mitochondrial function becoming impaired and energy reserves being reduced, which in turn would affect cellular osmoregulation, ion exchange, lipid peroxidation, and enzymatic activity, which regulate sperm motility [6,59,60]. Additionally, studies have demonstrated a significant correlation between mitochondrial membrane potential and fertilization rates of sperm in salmon trout and *Salmo salar*, implying that cryopreservation would affect the sperm motility and fertilization potency via disrupting mitochondrial function [61,62].

According to a study in *Silurus lanzhouensis*, its sperm is of a single-flagellum type with unique structures [63]. The sperm has three parts: the head, the middle, and the tail, with no acrosomes. The centrioles are perpendicular to each other in a T-shape, and the flagella are of typical “9 + 2” microtubule structures. The structure of sperm in *Silurus lanzhouensis* were obviously damaged during cryopreservation, including membrane (cytoplasmic membrane and chromatin membrane showing damage, wrinkling, vascularization, or overall shedding, and the gap between the nuclear membrane and the plasma membrane enlarging), nuclear deformation, and mitochondrial content leakage [63]. The observed structural changes post-cryopreservation, including head swelling, tail shortening, and midpiece deformities, are consistent with cryodamage mechanisms such as osmotic stress and ice crystal formation [63]. Species-specific responses, such as midpiece elongation in *Glyptosternum maculatum* in this study, highlighted varying cryotolerance levels, and were possibly due to differences in the membrane composition or structural robustness of different fish sperm. These findings underscore the need for optimizing cryopreservation protocols for different fish species, including using optimal cryoprotectant formulations and cooling rates, to minimize structural damage and effectively preserve fish sperm. Further studies should explore the correlation between these morphological changes and post-thaw motility or fertilization capacity to improve the outcomes of cryopreservation. Meanwhile, regular holes appeared on the surface of the heads of cryopreserved sperm in *Glyptosternum maculatum*; this is speculated to be attributed to the disappearance of some proteins or other macromolecules in cell membranes, but, to address this issue, more extensive investigations are needed in future study.

Although the cryopreservation method was optimized through the extensive investigations on cryomedium, freezing procedures, and related factors, there are still limitations in our study. For example, the potential impact of fish age on sperm cryopreservation was not evaluated, and fish age usually influences sperm quality and cryotolerance. In some species, older fish might have sperm with reduced motility and viability even before cryopreservation, thus affecting the cryopreservation process and the overall outcomes [64]. Seasonal change should also play a crucial role in sperm cryopreservation. The physiological state of fish, including sperm quality and motility, would be different in different seasons in a year. In most fish species, sperm production and quality are regulated by seasonal factors such as temperature, photoperiod, and food availability [65]. In our study, the sperm were collected in only one or two seasons, so the seasonal factors were ignored.

To develop more practicable methods of sperm cryopreservation in other fish species, further extensive investigations are needed in the future. It is necessary to test new combinations of cryoprotectants. Although, in this study, some common cryoprotectants were explored, there are numerous substances and combinations that need to be tested, such as the novel synthetic polymers or natural compounds with cryoprotective properties. These new cryoprotectants or combinations might provide better protection to sperm during the freezing and thawing process, potentially improving sperm viability and reducing cryoinjuries.

More importantly, the cryopreservation protocols could be applied in many more fish taxonomic groups. In this study, we only developed the cryopreservation protocol for three fish species, including koi carp, Ya fish, and *Glyptosternum maculatum*, and more effective cryopreservation strategies applicable to various fish species need to be developed in the future studies. Additionally, to elucidate the molecular mechanisms underlying sperm cryoinjuries, more investigations are also needed in the future.

## 4. Materials and Methods

### 4.1. Fish and Samples

Ten koi carp (SL: 29.58 ± 2.06 cm, BW: 453 ± 36.09 g) and ten Ya fishes (SL: 29.58 ± 2.06 cm, BW: 453 ± 36.09 g) were bought from the market (Snow Lake Fish Brand Headquarter at Fisherman’s Bay Wharf) and maintained in the experimental station of Southwest University, Chongqing, China. Before the experiments, five healthy males were selected and induced to spawn using carp pituitary extract. After 24 h, sperm was gently squeezed out and collected through abdominal massage. Subsequently, sperm with high motility (>80%) were mixed for further analysis (Figure S1).

*Glyptosternum maculatum* (SL: 29.58 ± 2.06 cm, BW: 453 ± 36.09 g) were maintained at the experimental station of the Institute of Fisheries Science, Tibet Academy of Agricultural and Animal Husbandry Sciences, Lhasa, China. The testes of eight *Glyptosternum maculatum* were surgically collected and the adherent tissue was dissected away; then, the testes were placed in a mortar. Testes were cut aseptically into pieces with scissors, then the released sperm were randomly aliquoted into three portions and gently agitated in extender solution. Then, the sperm were filtered through a cell strainer to collect samples for testing. Similarly, sperm with high motility (>80%) were mixed for further analysis.

All experiments were conducted in accordance with the Guide for the Animal Care and Ethics Committee of Southwest University (Chongqing, China). Furthermore, the Ethics Committee approved this research (Approval Code: IACUC-20210120-01; Approval Date: 10 January 2021).

### 4.2. Freezing Sperm

**Figure 7:**
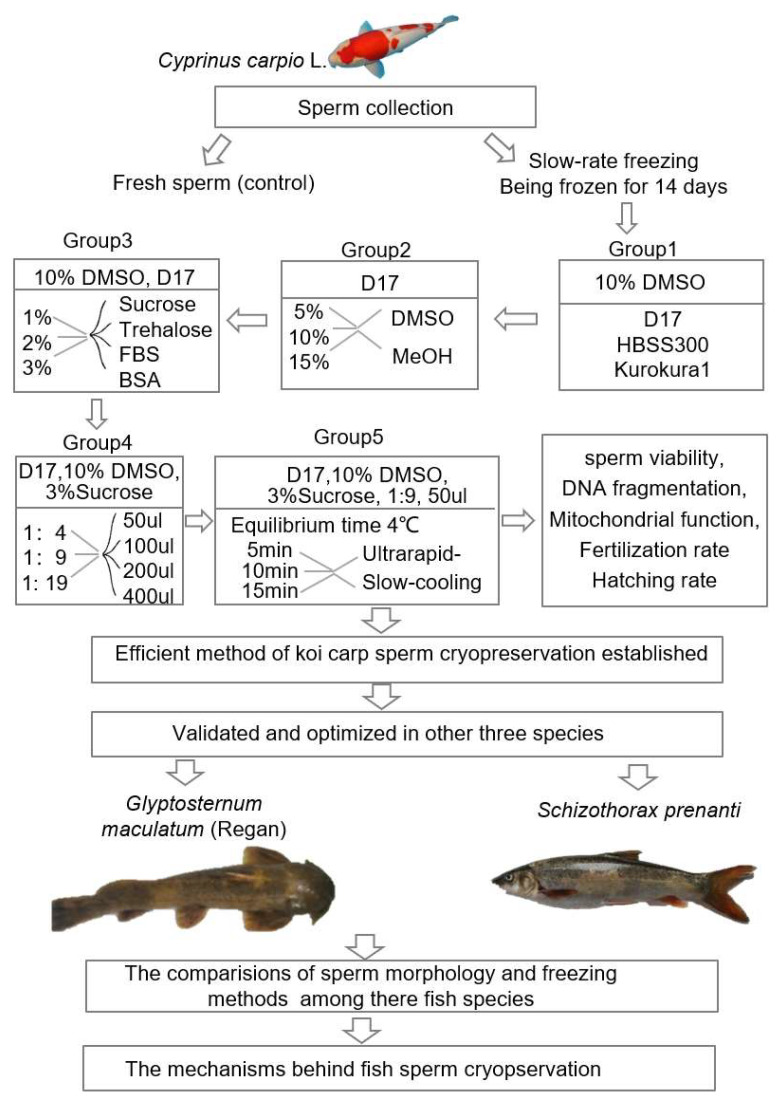
The flowchart of experiments. In this study, adult male koi carp (*Cyprinus carpio* L.), Ya fish (*Schizothorax prenanti*), and *Glyptosternum maculatum* (Regan) were collected from fish markets and farms. With the fresh sperm as control, the sperm viability was analyzed by AO/PI staining after a 14 d freezing in liquid nitrogen. Firstly, based on the analysis of sperm viability, the extender, cryoprotectant, volume, and cooling procedure were assessed and optimized in koi carp (Groups 1–5). And, secondly, the optimal protocol established was adopted to cryopreserve sperm in the two other fish species, Ya fish and *Glyptosternum maculatum* (Regan). Thirdly, the ultrastructure and freezing methods of sperm were comparatively analyzed among the fish species. All values are presented as the mean ± SEM calculated with the data of nine replicates. MeOH, methanol; DMSO, dimethyl sulfoxide; FBS, fetal bovine serum; BSA, bovine serum albumin.

To establish efficient sperm cryopreservation methods for koi carp, five experimental groups were sequentially designed. Different cryoprotectants, dilution ratios, mixture volumes, and cooling procedures were evaluated and optimized through assessing frozen–thawed sperm viability over 14–210 days (Figure 7), followed by a fertilization trial to determine the sperm quality. First, three extenders (D17, Kurokura1, HBSS300) were tested, with D17 (showing the highest sperm viability) selected for subsequent steps. Next, permeable cryoprotectants (DMSO and MeOH) at different concentration (5%, 10%, and 15%) were assessed. The reagent utilized in this study was purchased from Macklin Company. Subsequently, non-permeable agents (sucrose, D-trehalose, FBS, BSA) were evaluated at different concentrations (1%, 2%, and 3%). Throughout these trials, ultrarapid cooling was uniformly applied. In the fourth group, varied dilution ratios (Vol.sperm:Vol.cryomedium = 1:4, 1:9, and 1:19) and mixture volumes (50, 100, 200 and 400 μL) were tested. Finally, the fifth group investigated the equilibration times at 4 °C (5 min, 10 min, and 15 min) under both slow- and ultrarapid-cooling conditions. The steps of ultrarapid cooling are as follows: after mixing the sperm with the cryoprotectant, first place it at 4 °C for equilibration for 5 min; then, place it 10 cm above the liquid nitrogen for equilibration for 10 min; then, store it in liquid nitrogen. The steps of slow cooling are as follows: after mixing the sperm with the cryoprotectant, first place it at 4 °C for equilibration for 5 min; then, put it into a programmed cooling box (Labshark, cooling rate: −1 °C/min); then, place the programmed cooling box in a −80 °C refrigerator for 12 h; and, finally, store it in liquid nitrogen. The optimized protocol (50 μL mixture at 1:9 dilution, 10% DMSO + 3% sucrose in D17, ultrarapid cooling) was used to cryopreserve sperm in liquid nitrogen (LN) for 210 days. The fertilization rates of post-thaw sperm with fresh eggs were comparatively analyzed to determine the effects of long-term cryopreservation on the fertilization potency of sperm.

According to the koi carp sperm cryopreservation protocol, the protocol was further optimized and modified in Ya fish. Four experimental groups were sequentially designed to assess cryoprotectants, dilution ratio, mixture volume, and cooling procedures based on sperm viability after a 14-day freezing (Figure 7). Firstly, two cooling procedures (slow and ultrarapid cooling) were evaluated. The ultrarapid-cooling procedure was conducted as follows: the sperm was mixed with the cryoprotectant, and incubated at 4 °C for an equilibration of 5 min, then placed at ~10 cm above the liquid nitrogen for another equilibration of 10 min, and then stored in liquid nitrogen. The slow-cooling procedure was carried out as follows: the sperm was mixed with the cryoprotectant and incubated at 4 °C for an equilibration of 5 min, then put into a programmed cooling box (Labshark, cooling rate: −1 °C/min); then, the box was transferred to and kept in a −80 °C refrigerator for 12 h and, finally, stored in liquid nitrogen. Next, different permeable cryoprotectants (10% DMSO and 10% MeOH) were tested. The reagent utilized in this study was purchased from Macklin Company. Subsequently, two extenders (D17 and CCES2) were evaluated. Finally, several non-permeable agents (sucrose, D-trehalose, BSA, SMP) were tested at different concentrations (1%, 2%, and 3%). Throughout these trials, ultrarapid cooling was confirmed as the better option and adopted in the subsequent experiments. Additionally, dilution ratios (Vol.sperm:Vol.cryomedium = 1:4, 1:9, 1:19) and mixture volumes (50, 100, and 150 μL) were tested in the fourth group. The optimized protocol (50 μL mixture at 1:9 dilution, 10% DMSO + 3% SMP in D17, ultrarapid cooling) was used to cryopreserve sperm in LN for 14 days. In Ya fish, the cooling procedure adopted was the same as that for koi carp, and cryotubes were also stored in LN for a 14-day freezing.

The optimal protocol of sperm cryopreservation established in koi carp was applied in *Glyptosternum maculatum* with a little adjustment in volume (50 or 150 μL), dilution ratio, (1:4 or 1:9) and cryoprotectants (10% DMSO or 10% MeOH).

### 4.3. The Assessment of Sper’ Viability

Acridine orange/propidium iodine (AO/PI) staining was used to count the live and dead sperm following the description of our previous report [66]. Briefly, diluted sperm and AO/PI staining solution (Cat # CS2-0106, Vision CBA, Nexcelom, Lawrence, MA, USA) were mixed at a volume ratio of 1:1 and observed under the fluorescent microscope (Carl Zeiss, AxioObserver3, Oberkochen, Germany). The number of live sperm (green fluorescence) and dead sperm (red fluorescence) were counted under high magnification (400×) [66]. Each sample was examined twice, and at least three biological samples were analyzed per group.

Sperm Viability (%) = (number of live sperm)/(total number of sperm) × 100%.

### 4.4. DNA Damage

The DNA damage was assessed though SCD test [67]. Briefly, fresh or frozen–thawed sperm were mixed with 1% low-melting-point aqueous agarose (to obtain a 0.7% final agarose concentration), pipetted onto a glass slide precoated with 0.65% standard agarose, and solidified at 4 °C. After being denatured by 0.08 N HCl, slides were sequentially transferred into lysis solutions (0.4 M Tris, 0.8 M DTT, 1% SDS, and 50 mM EDTA, pH 7.5 for 10 min at room temperature, followed by 0.4 M Tris, 2 M NaCl, and 1% SDS, pH 7.5 for 5 min at room temperature) to remove cells’ membranes and deproteinize nuclei. After being washed in Tris-borate-EDTA buffer (0.09 M Tris-borate and 0.002 M EDTA, pH 7.5) and dehydrated in sequential 70%, 90%, and 100% ethanol baths (2 min each), the slides were then stained by Diff-Quik reagent and observed under a microscope (Carl Zeiss, AxioObserver3, Oberkochen, Germany) with 400× magnification [67]. The SCD test was repeated three times and the DNA fragmentation index was calculated based on the following formula:

DFI = (number of sperm without or with small halo)/(total count of sperm) × 100%.

### 4.5. Mitochondrial Membrane Potential

The mitochondrial membrane potential was analyzed using Rhodamine 123 (Rh 123, Maokang, MX3208-5MG, Shanghai, China) [68]. This dye has the property of aggregating in the mitochondrial matrix and emitting green fluorescence when the mitochondrial membrane potential is high. In the experiment, 10 μM of Rhodamine 123 was used. The sample was mixed with this Rh 123 solution and incubated at 37 °C for 30 min. After that, the mixture was observed under a fluorescence microscope (Carl Zeiss, AxioObserver3, Oberkochen, Germany). Each test was repeated three times, and the high mitochondrial membrane potential rate was calculated according to the following formula [68]:

MMH = (number of spermatozoa with high mitochondrial membrane potential)/(total count of sperm) × 100%.

### 4.6. Fertilization and Hatching Rate

Three adult female koi carp were induced to spawn with carp pituitary extract. After ovulation (about 12 h), eggs were gently stripped into a 100 mL glass beaker. About 0.4 mL of eggs (100 oocytes) were placed in 3 Petri dishes separately for fertilization by frozen–thawed sperm. After being frozen in LN for 14 d and 210 d, cryotubes were thawed and mixed with the newly stripped eggs in Petri dishes, and 10 mL of pond water was added into the Petri dish to activate the sperm. The sperm-to-egg ratio was 1 × 10^5^ sperm per egg. After an incubation of 10 min at room temperature (RT), the eggs were briefly rinsed and transferred to a 200 mL glass beaker containing 150 mL water (RT), oxygenated with an air pump. At ~9 h post-fertilization (hpf), a sample of ~100 eggs were randomly collected from each beaker to determine the fertilization rate (number of fertilized eggs reaching the blastula stage/initial number of eggs) using an optical microscope, and then hatching rate (number of hatched larvae/number of eggs) was observed and calculated (Figure S2).

### 4.7. Electron Microscopy

Sperm samples were aseptically collected and kept at 4 °C to protect cells’ viability. The sperm was fixed with a fixative solution (e.g., 1.25% glutaraldehyde in 0.1 M phosphate buffer, pH 7.4), incubated at 4 °C for 1–2 h, then centrifuged at 300 g for 10 min, and the pellet of sperm was washed three times to remove excess fixative. The sample was gradually dehydrated with ethanol at 30%, 50%, 70%, 90%, 100%, incubated for 10 min at each concentration, and underwent the 100% ethanol step twice. The sample was air-dried in a fume hood and attached to SEM stubs with conductive adhesive (e.g., carbon tape), then coated with gold or platinum using a sputter coater. The prepared sample was loaded into the SEM chamber. The ultrastructure and morphology of sperm were observed and comparatively analyzed among three fish species by ZEISS EVO 10. The microscope settings such as voltage, working distance, and detector were adjusted to achieve high-resolution imaging. High-resolution sperm images were taken and analyzed to show sperm morphology, surface features, and ultrastructure.

### 4.8. Statistical Analysis

The data were shown as mean ± SD. The significance of differences in sperm quality among the groups was assessed by a one-way analysis of variance (ANOVA) in conjunction with Duncan’s multiple range test using SPSS 20.0 (SPSS Inc., Chicago, IL, USA). An extreme significance level of <0.05 was set and used throughout all statistical analyses. In each experimental group, three biological samples were used and each sample was examined at least three times; all experiments were repeated at least twice. That is to say, each value is calculated from 9 sample data (n = 9).

## 5. Conclusions

In conclusion, this study successfully established efficient sperm cryopreservation methods for three fish species: koi carp, Ya fish, and *Glyptosternum maculatum*. The viability of frozen–thawed sperm in these species ranged from 45.39 ± 4.70% to 70.45 ± 2.23%. In koi carp, the fertilization rate of cryopreserved sperm reached up to 63.03 ± 1.36% and was only slightly affected by the cryopreservation time. Cryopreservation was found to cause significant injuries to sperm, including reduced cell viability, compromised membrane integrity, damaged DNA integrity, decreased mitochondrial activity, and changes in ultrastructure such as head swelling and tail shortening. These findings lay a foundation for developing more practical protocols for sperm cryopreservation in different fish species, which is crucial for the conservation of fish genetic diversity.

## Figures and Tables

**Table 1:** Effects of cryopreservation on the ultrastructural morphology of sperm.

	Characteristics	Head Length (μm)	Head Width (μm)	Midpiece Length (μm)	Midpiece Width (μm)	Tail Length (μm)	Tail Width (μm)
Sperm Specimens							
Koi carp (*Cyprinus carpio* L.)	Fresh	1.93 ± 0.027	1.62 ± 0.17	0.63 ± 0.14	1.19 ± 0.25	37.49 ± 3.58	0.36 ± 0.055
	Frozen	2.25 ± 0.32	1.96 ± 0.36	0.59 ± 0.24	1.00 ± 0.20	28.48 ± 7.30	0.29 ± 0.029
*Glyptosternum maculatum* (Regan)	Fresh	1.44 ± 0.16	1.57 ± 0.28	0.58 ± 0.12	1.56 ± 0.43	24.81 ± 2.75	0.28 ± 0.013
	Frozen	1.89	2.00	0.63	1.15	5.56	0.36
Ya fish (*Schizothorax prenanti*)	Fresh	2.33 ± 0.15	2.24 ± 0.13	0.58 ± 0.13	1.20 ± 0.28	8.19 ± 2.49	0.26 ± 0.042
	Frozen	2.71 ± 0.054	2.65 ± 0.28	1.27 ± 0.58	1.60 ± 0.70	5.60 ± 1.77	0.35 ± 0.11

## Data Availability

All data have been included in the manuscript.

## Supplementary Materials

The following supporting information can be downloaded at: https://www.mdpi.com/article/10.3390/ijms26104648/s1.
