# Supplementary material for: Developing Efficient Methods of Sperm Cryopreservation for Three Fish Species (Cyprinus carpio L., Schizothorax prenanti, Glyptosternum maculatum)
Source: Int J Mol Sci. 2025 May 13;26(10):4648. doi: 10.3390/ijms26104648 (PMC12111003; doi:10.3390/ijms26104648)
Supplement: Supplementary file 1 [file ijms-26-04648-s001.zip › ijms-3577685-supplementary.pdf]

Table S1. The protocol of sperm cryopreservation optimized in Koi carp.

Factors	Group	Sperm viability (%)
Extenders	Fresh sperm	97.62±0.40[a]
	D17	42.18±4.29[b]
	Kurokura1	34.32±4.85[b]
	HBSS300	14.19±4.46[c]
Permeable cryoprotectant	Fresh sperm	97.62±0.40[a]
	5% DMSO	28.31±8.73[bc]
	10% DMSO	42.18±4.29[b]
	15% DMSO	14.19±6.96[bc]
	5% MeOH	11.47±5.58[d]
	10% MeOH	18.56±3.35[d]
	15% MeOH	13.06±5.24[d]
Nonpermeable cryoprotectant	Fresh sperm	81.73±7.26[a]
	No addition	25.11±3.35[bc]
	1% Sucrose	21.45±5.39[cde]
	2% Sucrose	23.13±9.92[cd]
	3% Sucrose	35.62±1.7[b]
	1% Trehalose	31.94±8.55[bc]
	2% Trehalose	11.53±0.97[def]
	3% Trehalose	2.01±1.04[f]
	1% BSA	2.99±0.38[cde]
	2% BSA	2.33±0.58[f]
	3% BSA	3.28±0.06[f]
	1% FBS	10.46±2.46[ef]
	2% FBS	4.65±1.27[cde]
	3% FBS	9.09±0.93[e]
Dilution ratio and volume	1:4/50μl	26.37±1.76[bcd]
	1:4/100μl	28.94±2.09[bc]
	1:4/200μl	20.17±7.08[de]
	1:4/400μl	2.56±0.52[f]
	1:9/50μl	41.98±4.02[a]
	1:9/100μl	30.74±1.93[b]
	1:9/200μl	20.72±3.77[cde]
	1:9/400μl	3.08±0.56[f]
	1:19/ 50μl	32.16±5.85[b]
	1:19/ 100μl	40.72±0.66[a]
	1:19/200μl	31.98±0.47[b]
	1:19/400μl	17.27±2.94[e]
Cryopreservation protocol and Equilibrium time at 4°C	Ultrarapid-cooling/5min	48.62±5.62[a]
	Ultrarapid-cooling/10min	45.75±5.61[a]
	Ultrarapid-cooling/15min	41.70±5.20[ab]
	Slow freezing/5min	40.48±3.57[ab]
	Slow freezing/10min	29.17±3.89[b]
	Slow freezing/15min	39.79±8.10[ab]

The viability of koi carp sperm was analyzed with AO/PI staining. Effects of different extenders, permeating cryoprotectants, non-permeating cryoprotectants, volumes, dilution, cryopreservation protocol and equilibrium time on sperm viability. All values are presented as the mean ± SEM calculated with the data of nine replicates. Different letters indicate the significance of differences ($p < 0.05$).

Table S2. The efficiency of the optimized protocol in different fish specie.

Species	Sperm viability (%)	
	Fresh	Frozen
Koi carp (*Cyprinus carpio* L.)	93.54±2.47	63.23±1.36
Ya-fish (*Schizothorax prenanti*)	81.34±1.75	27.60±2.96
Glyptosternum maculatum (Regan)	80.46±4.33	45.39±4.7

The established optimal protocol was adopted to cryopreserve sperm from another batch of koi carp and other two fish species, Ya fish, *Glyptosternum maculatum* (Regan). With the fresh sperm as control, the sperm viability was analyzed by AO/PI staining after a 14d-freezing in liquid Nitrogen. All values are presented as the mean ± SEM calculated with the data of nine replicates.

Table S3. The efficiency of the optimized protocol in different fish species.

Factors	groups	Sperm viability (%)
Cryopreservation protocol	Fresh sperm	85.07±1.88[a]
	Ultrarapid-cooling	27.41±4.85[b]
	Slow freezing	22.01±6.91[b]
	Fresh sperm	85.07±1.88[a]
Permeable cryoprotectant and extenders	Fresh sperm	90.37±1.41[a]
	DMSO/ D17	26.00±2.10[b]
	MeOH/ D17	15.01±4.66[c]
	Glycerol/ D17	17.28±2.41[bc]
	DMSO/ CCES2	18.32±3.31[bc]
	MeOH/ CCES2	10.00±3.89[c]
	Glycerol/ CCES2	16.86±5.13[c]
Nonpermeable cryoprotectant	Fresh sperm	80.06±3.58[a]
	Control	22.04±4.44[h]
	1% Sucrose	34.41±6.32[g]
	2% Sucrose	39.73±3.28[efg]
	3% Sucrose	21.24±2.07[h]
	1% Trehalose	34.34±7.08[g]
	2% Trehalose	37.87±3.78[fg]
	3% Trehalose	32.37±2.49[g]
	1% BSA	44.41±7.46[efg]
	2% BSA	46.64±4.66[def]
	3% BSA	48.50±3.48[cde]
	1% SMP	54.79±3.03[cd]
	2% SMP	57.13±1.40[bc]
	3% SMP	65.62±3.78[b]
Dilution ratio and Volume	1:4/50μl	55.40±6.39[abc]
	1:9/50μl	70.45±2.23[a]
	1:19/50μl	68.78±1.45[ab]
	1:4/100μl	52.06±11.17[cd]
	1:9/100μl	30.55±3.13[e]
	1:19/100μl	54.33±2.77[bc]
	1:4/150μl	55.16±8.35[abc]
	1:9/150μl	34.24±2.38[e]
	1:19/150μl	38.95±9.02[de]

The established optimal protocol was adopted to cryopreserve sperm from another batch of koi carp and other two fish species, Ya fish, *Glyptosternum maculatum* (Regan). With the fresh sperm as control, the sperm viability was analyzed by AO/PI staining after a 14d-freezing in liquid Nitrogen. All values are presented as the mean ± SEM calculated with the data of nine replicates. Different letters indicate the significance of differences ($p < 0.05$)

Figure S1

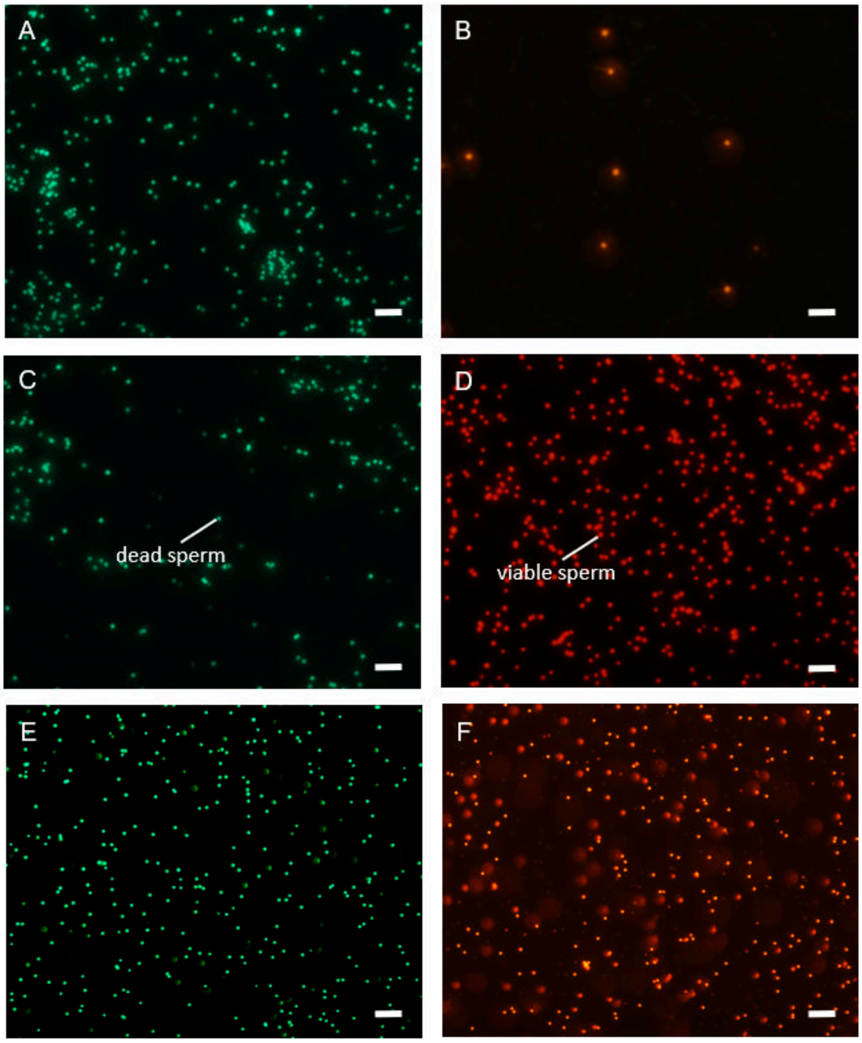

Figure S1 The viability of koi carp sperm (AO/PI staining)
A, C and E, AO staining for the membrane integrity of live sperm. B, D and F, PI staining for the dead sperm (Red). A-B, Fresh sperm untreated. C-F, The frozen-thawed sperm after a 7-day-freezing in liquid nitrogen via the ultrarapid-cooling procedure. C-D, the sperm was frozen with 100 µL cryomedium containing 10% DMSO dissolved in D17. E-F, Sperm diluted by D17 (50 µL, 1:9) and frozen with the cryomedium containing and 3% of sucrose. Scale bars, 50 µm.

Figure S2

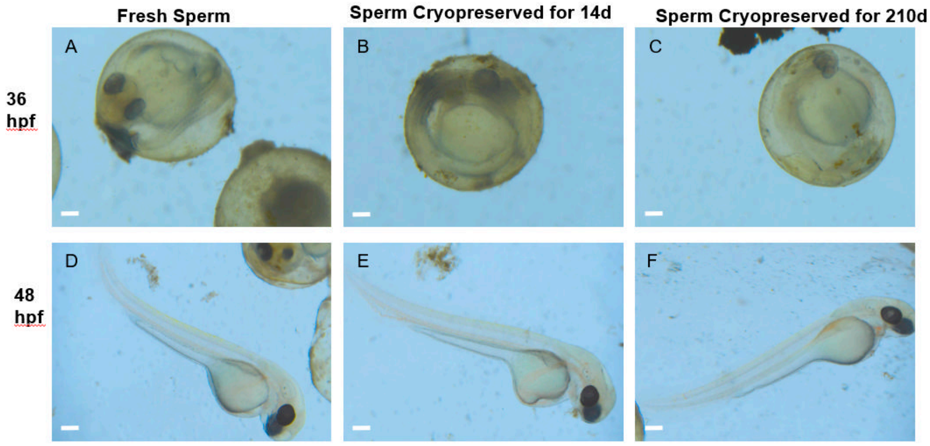

Fig. S2 The embryonic development of fertilized eggs

(A-C) The embryos at 36 hpf; (D-F) The fries at 48 hpf. hpf, hour after fertilization. Scale bars, 200 μm.
